# A Clinician's Guide to Sharing Data for AI in Ophthalmology

**DOI:** 10.1167/iovs.65.6.21

**Published:** 2024-06-12

**Authors:** Nayoon Gim, Yue Wu, Marian Blazes, Cecilia S. Lee, Ruikang K. Wang, Aaron Y. Lee

**Affiliations:** 1 Department of Ophthalmology, University of Washington, Seattle, WA, United States; 2 The Roger and Angie Karalis Retina Center, Seattle, Washington, United States; 3 Department of Bioengineering, University of Washington, Seattle, WA, United States

**Keywords:** data sharing, data license, differential privacy, federated learning, trusted research environments

## Abstract

Data is the cornerstone of using AI models, because their performance directly depends on the diversity, quantity, and quality of the data used for training. Using AI presents unique potential, particularly in medical applications that involve rich data such as ophthalmology, encompassing a variety of imaging methods, medical records, and eye-tracking data. However, sharing medical data comes with challenges because of regulatory issues and privacy concerns. This review explores traditional and nontraditional data sharing methods in medicine, focusing on previous works in ophthalmology. Traditional methods involve direct data transfer, whereas newer approaches prioritize security and privacy by sharing derived datasets, creating secure research environments, or using model-to-data strategies. We examine each method's mechanisms, variations, recent applications in ophthalmology, and their respective advantages and disadvantages. By empowering medical researchers with insights into data sharing methods and considerations, this review aims to assist informed decision-making while upholding ethical standards and patient privacy in medical AI development.

Data diversity and quality are crucial in harnessing the full potential of AI models, because their effectiveness depends on the breadth and depth of the data. Especially in medical AI, diverse datasets that encompass a wide range of patient ethnicities, demographics, and clinical environments are essential in creating robust and generalizable AI solutions. However, data sharing in healthcare has significant obstacles and concerns. The main challenge is the inherent tension between open collaboration versus the sensitive nature of healthcare data. Issues related to patient privacy, security, data and model ownership, access rights, licensing, and a myriad of regulatory and ethical considerations further complicate the data sharing process.

Clinicians, who are at the forefront as primary data generators, play a pivotal role within the medical AI ecosystem. Their decisions on data sharing, often dictated by varying levels of security and openness, have far-reaching impacts on the development and effectiveness of AI applications in medicine. This article is intended to empower medical researchers with knowledge and insights to make informed decisions to contribute to the advancement of AI in ophthalmology while upholding the standards of patient privacy and ethical practice.

In this review, we present a comprehensive examination of data sharing methods currently in the medical field, covering the spectrum from traditional to nontraditional approaches. The traditional methods involve transfer of data itself between users, such as openly sharing public datasets, and sharing data based on data use agreements. The newer nontraditional approaches focus on addressing security and privacy considerations by eliminating the need for transferring data between users. These methods are categorized into three types: 1. Sharing derived datasets by using techniques that enhance de-identification (e.g., differential privacy and synthetic data); 2. Bringing users to data in a secure location, without needing to transfer data (e.g., trusted research environments); and 3. Bringing models to data, eliminating the need to move data and users (e.g., model-to-data approach and federated learning). For each method, we discuss its mechanism, popular variations, recent use cases in the field of ophthalmology, and the advantages and disadvantages.

## Traditional Approaches to Share Data

### Publicly Available Clinical Datasets

**Figure 1.**
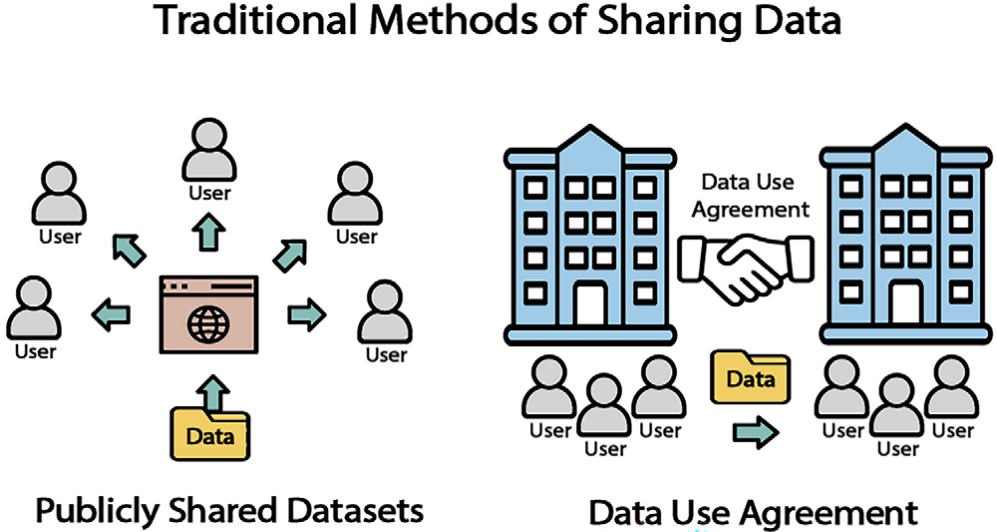
Publicly shared datasets and data use agreement.

Publicly available clinical datasets are important resources for the medical research community as they are free and easily accessible. In this review, publicly shared datasets are defined as data that is accessible on the internet by unspecified users through a simple process, such as directly downloading files from a source or creating an account with minimum personal information like an email address (Fig. 1). By removing barriers to access, these datasets help democratize medical research, allowing broader participation. The open nature of these datasets can foster transparency in research, enabling replication of studies, and encouraging a wide array of analyses and innovations.^1^

Methods for sharing data publicly often have varying terms for allowing users to download, copy, and distribute data. These methods will be discussed in the review from most open to most restrictive. Public domain dedication is the most open option and is based on the assumption that people or entities cannot “own” natural facts. Public domain dedication removes copyright ownership, allowing others to use the data without restriction or need for attribution of authorship.^2^ Creative Commons Zero Public Domain Dedication falls in this category.^2^ Creative Commons License (CCL) similarly allows others to download and distribute the work but is more restrictive, because all types of CCLs allow authors to maintain copyright ownership.^2^ CCLs require users to acknowledge the original creator but can have a range of additional conditions (Table 1).

**Table 1.** Creative Commons Zero and Creative Commons License Variations

Category	License Name	Abbreviations	Requirements for Users
Creative Commons Zero (CC0)	Not applicable	CC0	• Public Domain Dedication • No need to acknowledge the original creator • No restrictions on derivatives or commercial use
Creative Commons License (CCL)	Creative Commons Attribution	CC-BY	• The original acknowledged (BY)
	Creative Commons Share-Alike	CC-SA	• The original creator is acknowledged • Work derived from the original uses the same license terms (SA)
	Creative Commons Attribution-NoDerivs	CC BY-ND	• The original creator is acknowledged (BY) • Creating derivative works based on original work is restricted (ND)
	Attribution-NonCommercial	CC BY-NC	• The original creator is acknowledged (BY) • Commercial use is restricted (NC)
	Attribution-NonCommercial-ShareAlike	CC BY-NC-SA	• The original creator is acknowledged (BY) • Work derived from the original uses the same license terms (SA) • Commercial use is restricted (NC)
	Attribution-NonCommercial-NoDerivs	CC BY-NC-ND	• The original creator is acknowledged (BY) • Commercial use is restricted (NC) • Creating derivative works based on the original work is restricted (ND)

Ophthalmic datasets span a range of imaging and data types. The most common types of ophthalmic data, in decreasing order, are fundus images, optical coherence tomography (OCT), and OCT angiography (OCTA) scans, external eye photographs, in vivo confocal microscopy images, and videos.^3^ Datasets can also be categorized based on the specific eye diseases they cover. This includes datasets focused on healthy eyes, which serve as controls or baselines, as well as those dedicated to prevalent eye conditions. The most common types of eye disease datasets, in decreasing order, are healthy eyes, diabetic retinopathy, glaucoma, age-related macular degeneration (AMD) and hypertensive retinopathy.^3^ This disease-specific categorization is crucial for researchers focusing on particular eye conditions, facilitating targeted studies and advancements in diagnosis and treatment.

In the realm of ophthalmology, several open datasets have played a crucial role in advancing research. These datasets include the diabetic retinopathy detection challenge dataset (the EyePACs dataset) from Kaggle,^4^ the CASIA Iris Images,^5^ the STARE Dataset (Structure Analysis of the Retina),^6^ Harvard's GDP500, GD100, and GF3300., EF30k, FairSeg10k,^7^ Messidor,^8^ Duke's OCT datasets,^9^ and the OLIVES Dataset.^10^

However, there are also notable drawbacks to open datasets. One of the primary concerns is the possibility of inadequate data privacy protection. Despite de-identification efforts, there is always a risk of re-identification, especially as data analysis techniques become more sophisticated.^11^ This raises concerns for patient privacy and ethical standards in research, especially in the realm of eye-related images such as iris, external eye, and retina. Ongoing debates persist about the sensitive health information that can be publicly shared in these contexts, necessitating clear boundaries. Another challenge is data fragmentation and the potential for the misuse of data. When datasets are widely accessible, there is a risk that they may be used inappropriately or taken out of context, leading to inaccurate conclusions or misuse. Another important aspect is ensuring the quality of data. Given that machine learning models heavily depend on accurate labeling, any inaccuracies can directly impact model performance. Numerous studies have highlighted this concern and suggested various methods for enhancement of labeling accuracy.^12^^,^^13^ Considering the wide usage of publicly accessible datasets in model training, upholding a high bar for label accuracy is of importance. These disadvantages and obstacles highlight the need for careful management and regulation of publicly available clinical datasets to ensure they serve their intended purpose effectively and ethically.

### Sharing Data Based on Data Use Agreement

Another popular traditional model for sharing health care data is with a Data Use Agreement (DUA). A DUA is a legal contract between two entities: the data owner and the data receiver; the DUA specifies the details regarding how the data can be used, requirements that the data receiver should satisfy (e.g., affiliated institution, faculty status, IRB approval), limitations on data use (e.g., data removal after a certain period, no commercial use, citing the original source when work using the data is disseminated).^14^ In general, it is recommended that a DUA is negotiated whenever transferring non-public data that comes with specific limitations on its utilization.

Regarding medical data, based on the level of de-identification of patients, data can be categorized into three categories. The first category is identifiable medical data that include any information that identifies patients, such as names, all geographical subdivisions more specific than a state, all dates directly related to an individual (e.g., birth date, admission/discharge date, date of death), for patients over 89, birth year is also considered identifiable medical data, phone/fax/medical record/health plan beneficiary/account/license/vehicle/device serial numbers, email/IP addresses, URLs, biometric identifiers (e.g., finger and voice prints), full face photographic images, and any other unique identifiers unless permitted and specified by the Health Insurance Portability and Accountability Act of 1996 (HIPAA) Privacy Rule for re-identification.^15^^,^^16^ Sharing identifiable data requires individual patient consent and cannot be shared using DUAs alone. The second category is indirectly identifiable medical data, commonly referred to as a “Limited Data Set.” In a Limited Data Set, identifiers unique to patients have been removed but still contain sensitive information that can indirectly help identify patients, such as city, town, full zip code, and dates such as date of admission/discharge or date of birth (using just year may be recommended).^17^ For sharing Limited Data Sets, individual consent from patients is not required, but a DUA must be in place before sharing. The third category is fully de-identified data that is considered to be of minimal or no risk of re-identification and not governed by HIPAA. This category does not require either patient consent or DUA for sharing data. The two methods for de-identification under HIPAA are Safe Harbor and Expert Determination.^15^ The Safe Harbor method requires the removal of all 18 direct identifiable data from the first category and the removal of actual knowledge that could be used alone or in combination with other information to identify an individual. The Expert Determination method requires a qualified expert to evaluate the risk of re-identification and document the analysis to confirm that the risk is minimal.

DUAs have their own advantages and drawbacks. DUAs provide legal protection by clearly defining data rights, responsibilities, and restrictions, which can help prevent future misunderstandings and disputes. They can improve data security by ensuring secure handling of sensitive information in compliance with data protection laws, reducing the risk of violations. They aid in regulatory compliance, helping organizations follow data privacy regulations like HIPAA. Furthermore, DUAs establish clear terms for data access, ownership, sharing, and destruction, fostering transparency and accountability, and they can also mitigate risk by outlining consequences for noncompliance and data misuse. However, DUAs have some disadvantages. Drafting and negotiating DUAs can be complicated and time-consuming, particularly when parties have differing interests, which can cause significant delays in research projects and collaborations. Some DUAs may impose restrictions, limiting the flexibility of researchers or organizations to use data for future projects. Additionally, legal consultation for DUAs may create additional costs, and the presence of a DUA may discourage potential collaborators who find the terms overly restrictive and overwhelming. DUAs can pose significant hurdles for researchers operating in low-resource environments with limited infrastructure and capacity to navigate the DUA process. This can worsen disparities in accessing data, worsening the issues of inequity. Therefore, thorough consideration is essential when implementing DUAs to weigh their advantages and disadvantages effectively.

## Sharing Datasets Securely

### Differential Privacy

Differential Privacy is a method designed to safeguard the privacy and security of an individual's data within datasets while allowing extraction of useful information for the desired data analysis.^18^ It is structured to preserve patterns and characteristics at the group level rather than to preserve identifiable information about specific individuals by using data perturbation techniques such as adding controlled noise directly to the data.^19^ These methods can effectively preserve the overall integrity and global information of the dataset, while substantially minimizing the risk of disclosing details about individual data points. This balance between maintaining the utility of data for different purposes such as research and upholding privacy standards for individuals makes differential privacy a particularly valuable tool.

The implementation and general workflow of differential privacy involves a series of steps, beginning with identifying the specific data requiring protection and establishing clear privacy goals. Following this, privacy parameters are chosen, such as ε (epsilon), one of the most commonly used privacy parameters. ε controls how much the presence or absence of a single datapoint can affect the outcomes of a privacy-protection mechanism.^20^ Epsilon quantifies the change in query results when executed on two nearly identical databases, one of which has just one less data point than the other. A smaller epsilon value indicates better privacy protection, as it shows that the query results are minimally affected by the addition or removal of a single data entry.

Definition of differential privacy could be described by this equation^21^:

$$\mathrm{Pr}\left[M\left(x\right)\in S\right]\leq \exp\left(ɛ\right)\;\mathrm{Pr}\left[M\left(y\right)\in S\right]+\delta .$$

This is a condition specifying when a randomized algorithm M is considered differentially private. This inequality shows that the probability of the algorithm outputting a result in a group of potential output of M,S, when the input for an algorithm M is x, which is at most e^ε (epsilon) times the probability of outputting S when the input for the same algorithm M is y, plus a small additional probability δ (delta), which represents a privacy breach. This ensures that the presence or absence of any individual's data in the input has limited impact on the probability distribution of the output and therefore protects privacy.

Based on the chosen epsilon, a controlled amount of noise is added to the data, which masks the contributions of individual data points and prevents the inference of private information from the aggregate data. Differential privacy typically requires a trusted curator that can manage this process on the original dataset.^21^

In the context of epsilon based differential privacy implementation, different methods could be used for noise addition, such as the Laplace mechanism, the exponential mechanism, randomized response, and the Gaussian mechanism.^21^ The Laplace mechanism adds noise according to the Laplace distribution. The exponential mechanism introduces controlled randomness into the response selection process to find the right balance of privacy protection and the utility of the chosen response. Randomized response can be used for survey data because it introduces randomness into individual responses making it challenging to isolate an individual's true response; the Gaussian noise mechanism, on the other hand, adds noise following a Gaussian distribution. Another approach to categorize differential privacy is based on the scale of implementation: local differential privacy and global differential privacy. Local differential privacy involves adding noise to the data at the individual level, where each data point is independently perturbed before any analysis or aggregation.^22^ This approach highly enhances privacy protection but can result in higher noise levels in the aggregated data. Global differential privacy, in contrast, perturbs the output of aggregated queries from the database, offering a balance between privacy protection and data utility.

In the field of ophthalmology, the application of differential privacy has been progressively explored through several studies. A 2019 study applied differential privacy to eye-tracking data, focusing particularly on static heatmaps.^23^ The article outlines a situation in which an unauthorized party gains access to an individual child's eye-tracking heatmap data in a classroom setting, potentially disclosing diagnoses such as dyslexia. The authors showed that adding Gaussian noise could effectively ensure privacy, with the noise level being adjustable according to specific applications. The study also emphasized the necessity of extending privacy protection to other types of eye-tracking data, such as saccade velocity and attention allocation. Following this, a 2021 study found that eye-tracking data from virtual or augmented reality glasses can contain biometric information, and that eye movements can have consistent patterns that can become unique identifiers when analyzed over time.^24^ The study introduced two novel privacy methods, chunk-based and difference-based, for enhancing privacy of eye movement data.^24^ These methods aimed to reduce query sensitivity and temporal correlations and were evaluated on the MPIIDPEye and MPIIPrivacEye datasets. They proved to be more effective than standard techniques like the Laplace perturbation algorithm and Fourier perturbation algorithm, particularly in handling correlated data and ensuring personal privacy. Most recently, in 2023, a study evaluated de-identification of retinal scans using the Snow model and differential privacy on the BR-OPHTSET dataset.^25^ The unique and stable vascular pattern in retinal scans could serve as an individual identifier. Using the Snow model, pixel-level noise was added by arbitrarily reassigning pixel intensities. The study showed that sex de-identification was possible while preserving the performance of downstream tasks such as diabetic retinopathy classification. Together, these studies highlight the evolving role and efficacy of differential privacy methods in protecting patient data in ophthalmological research and applications.

Differential privacy's applicability extends across various domains, including machine learning, statistical analyses, and database queries. For example, Adnan et al.^26^ incorporated differential privacy to supplement their federated learning platform to provide quantitative bounds on the amount of privacy provided on medical imaging data. This versatility demonstrates its capacity to enhance privacy across different technological and research fields. Additionally, a significant advantage of differential privacy is the ability to control the extent of the privacy parameters. Users can adjust the level of privacy protection according to their needs, striking a balance between enhancing privacy protection and extracting meaningful insights from the data but this is also one of the limitations, in that it can be difficult to find the right balance between enhancing privacy protection and avoiding excessive data distortion. For example, Bagdasaryan et al.^27^ reported that accuracy of differential privacy drops in general, but the accuracy drops more for the under-represented classes. It is important to ensure that applying a differential privacy algorithm does not disproportionately put minorities at disadvantage. The researchers need to evaluate whether the accuracy, and minority group representation was affected to ensure equitable results. Too much noise can render the data less useful—advanced machine learning models or deep data mining tasks, which require detailed and precise data, can be hindered by excess noise—whereas too little may compromise privacy. Setting appropriate privacy parameters, such as the ε value, is another complex and challenging aspect of implementing differential privacy. Last, scalability is a significant challenge: Applying these privacy mechanisms to large datasets can be computationally expensive, posing limitations on the scalability of differential privacy solutions in handling massive volumes of data. These limitations highlight the need for ongoing research and development to optimize differential privacy for a wider range of applications and data sizes.

### Synthetic Datasets

A synthetic dataset is an artificially generated dataset specifically designed to mimic the characteristics, patterns, and structure of real data. This can be particularly valuable when patient confidentiality or sensitive information contained in data is a significant consideration.^28^ By simulating real-world data, synthetic datasets provide a safe and effective means to conduct extensive research and testing without risking patient privacy. In the past, synthetic data had been explored in optical security through the generation and subsequent recovery of random phase images.^29^ Recently, synthetic datasets have been used in machine learning applications to provide additional data and enrich representation, which has been shown to improve model performance in various cases, especially when original data are limited, such as in the case of rare diseases.^30^^,^^31^

**Figure 2.**
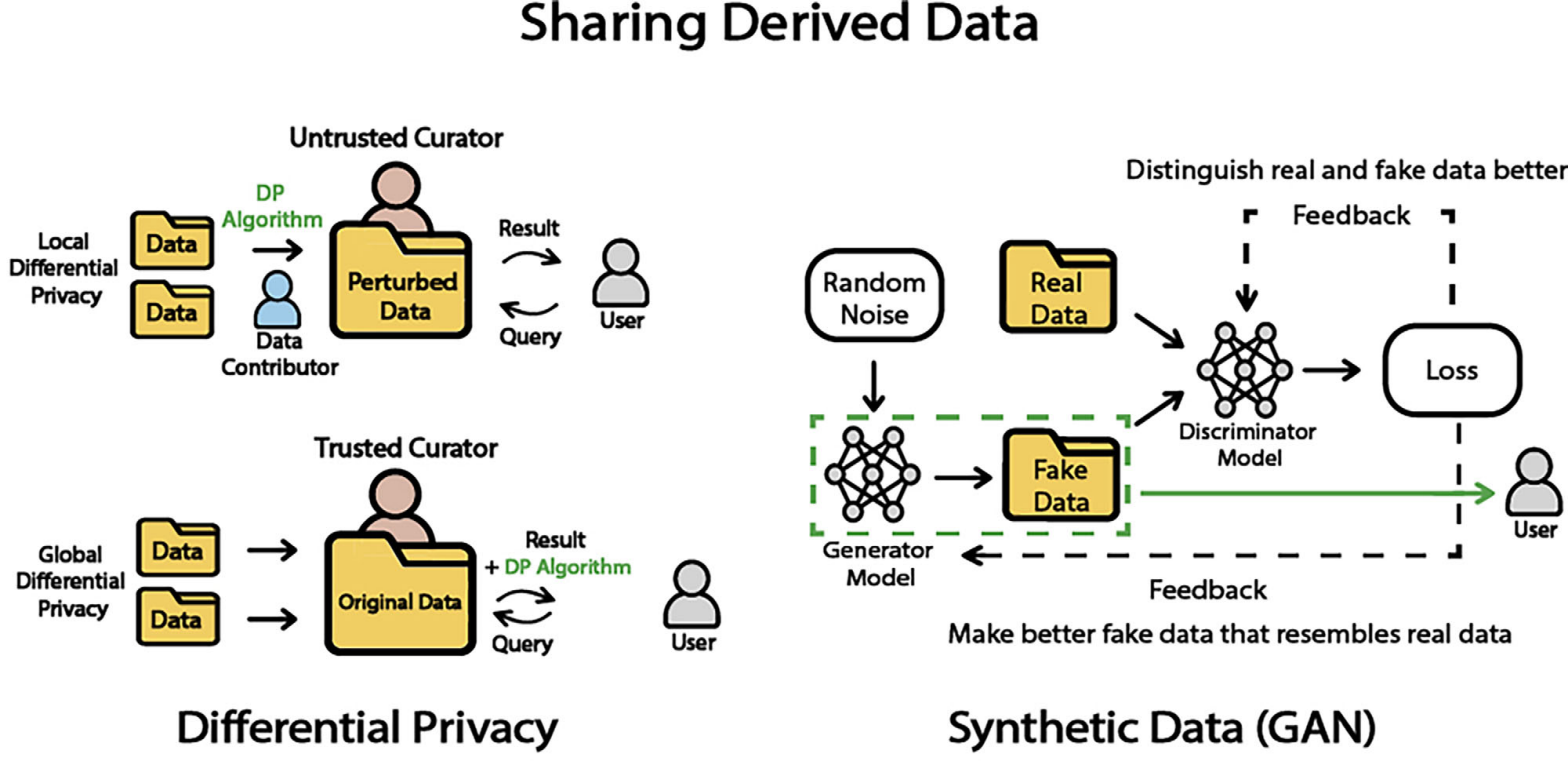
Differential privacy and synthetic data.

Synthetic datasets in the medical field can be categorized by data type (electronic health records, medical imaging, genomics, clinical trials data, etc) or by method of generation. Synthetic datasets can be generated using various methods, including statistical techniques like random sampling, multiple data imputation, and Bayesian bootstrap for maintaining statistical properties. Generative models like generative adversarial networks (GANs) and variational autoencoders, involve complex neural network training. As shown in Figure 2, GANs comprise a generator and a discriminator. During the training process, the generator improves its ability to produce data resembling real data, whereas the discriminator enhances its capacity to differentiate between real and fake data. This iterative process continues until the generator reaches a stage where it generates fake data that the discriminator can barely distinguish from real data. Additionally, there are knowledge-based approaches that leverage domain-specific knowledge to specify data requirements (e.g., patients diagnosed with X disease having a mutation in Y gene).

Although synthetic data inherently enhances patient privacy protection by being distinct from original patient data, its reported applications in ophthalmology primarily emphasize its role in improving model performance through data augmentation, thereby increasing the available training data. Menten et al.^32^ reported a pipeline for generating synthetic OCTA images with blood vessel segmentation labels. The authors used physiology-based modeling, such as retinal layer geometry, metabolic maps, graph-based modeling of angiogenesis, and physics-based image augmentation to simulate typical OCTA image characteristics such as flow projection artifacts, eye motion artifact, image noise, vitreous floater artifacts, and image noise to mimic the real OCTA images. This synthetic data aids in training vessel segmentation algorithms without the need for manual annotation. Danesh et al.^33^ proposed a method that used the Active Shape Model with a limited number of OCT images for training to create synthetic OCTs, delineate retinal boundaries, and point to abnormalities. The comparison of thickness maps showed that the synthetic dataset can be used as a statistically acceptable representative of the original dataset, suggesting that the proposed algorithm provided an augmentation method for OCT scans. Kim et al.^34^ reported generating realistic high-resolution color fundus images using style-based generative adversarial networks. They used the StyleGAN model, which learned to output high-quality resolution detailed images. Another study used StyleGAN2 to generate fundus autofluorescence images with realistic inherited retinal disease pathology. Both studies performed the Visual Turing Test to confirm that ophthalmologists struggled to distinguish between real and synthesized images, with a relatively random probability around 50%. Furthermore, a recent study used a generative adversarial network called GANSeg to develop an algorithm for segmenting intraretinal fluid and retinal layers in normal and pathological macular OCT images.^35^ Remarkably, the model's adaptability allowed it to generalize from training on one device (Heidelberg Spectralis) to other devices (Topcon 1000, Maestro2, Zeiss Plex Elite 9000), all without the need for labeled data from those devices.

In addition to improving model performance, synthetic datasets offer other advantages. The diversity in synthetic data is crucial for training algorithms to perform well across various scenarios and patient groups. Additionally, synthetic datasets are cost-effective compared to the use of real patient data or imaging, which can be expensive and logistically difficult to acquire. Another significant advantage is the ability to create more controlled datasets. Researchers can tailor synthetic datasets to include specific conditions or diseases for which real patient data is difficult to obtain bypassing the complexity of privacy regulations and ethical considerations.

However, notable drawbacks exist in using synthetic datasets. One major issue is that clinicians, regulatory bodies, and policy authorities may be skeptical about the reliability and validity of synthetic data. This skepticism can stem from concerns about how well these datasets mimic real-world scenarios and patient complexities. Synthetic datasets, despite their sophistication, cannot always fully capture the intricacies or nuances of real patient data. This limitation can lead to potential inaccuracies in research findings, posing a risk of misinformed conclusions. It is also important to acknowledge that there is no absolute guarantee that synthetic data cannot be used to re-identify individuals in the original set, especially with models that used real data for training. This aspect can potentially undermine their greatest advantage. It is essential to ensure that synthetic data generation processes are carefully monitored to mitigate biases, as observed in some contexts of generative AI, such as gender biases, depicting women as younger and smiling more in comparison to men in generative AI tools like Midjourney, Stable Diffusion, and DALLL·E.^36^^,^^37^ Zack et al.^38^ reported that GPT-4 showed racial and gender bias such as including diagnoses that stereotype certain races, ethnicities, and genders. Given the growing popularity of the usage of generative models, it is important to be aware of the biases and evaluate them and not blindly trust the results to reflect reality.

Furthermore, evaluating and validating synthetic datasets can be challenging. There are no established standards for objectively assessing how closely these datasets resemble real-world data. This lack of standardized evaluation criteria makes it difficult to determine the effectiveness and reliability of synthetic datasets in accurately reflecting real patient conditions, which is crucial for their acceptance and utility in medical research and practice.

## Bringing Users to Data in a Secure Location

### Trusted Research Environments

**Figure 3.**
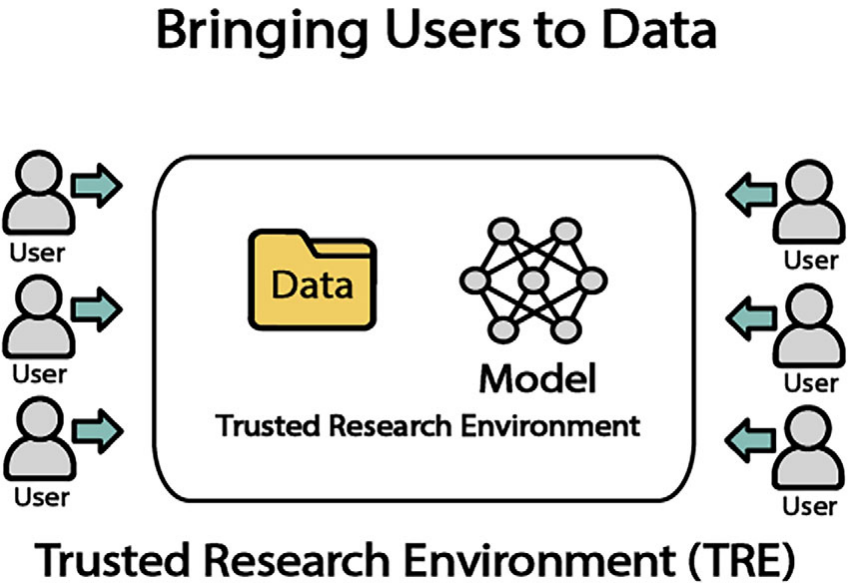
Trusted research environment.

In our data-centric era, organizations are seeking ways to harness computational power without compromising data security and privacy. In contrast to the traditional method of users downloading data from a central location, a growing trend is to provide trusted research environments (TRE) that have a secure computing environment and include both the datasets and tools for data analysis (Fig. 3). An example of a trusted research environment is an infrastructure set up where only authorized researchers have access to work with the protected data through a secure measure like multi-factor authentication. The servers within the TRE can include computing resources such as high-performing GPUs, and software needed for data analysis, such as R studio, python, and SAS. To safeguard the data, the datasets within the TRE can be configured to be read-only, which prevents alteration, download, or copying, or removal of data from the servers. Disabling internet connection, using end-to-end encryption, and limiting the lifespan of access to the environment can add another layer of security. The benefits of a TRE include adding a layer of security and privacy protection by avoiding the transfer of data from the data provider to the data user, as well as providing an equal ground for researchers to perform analysis, especially for those who have limited analysis-related resources at their institution.^39^ Typically a TRE requires a DUA between institutions that defines the terms and conditions regarding data access and the scope of use.

A review study by Kavianpour et al.^40^ collected and analyzed information from 73 TRE operators. Based on the responses, the majority of them were building their systems based on the TRE Green Paper by the UK Health Data Research Alliance, which describes the important five “Safes”: safe people, safe projects, safe setting, safe data, and safe outputs.^41^ Some of the well-known TREs in healthcare include services provided by Clinical Practice Research Datalink (CPRD),^42^ Electronic Data Research Innovation Services (eDRIS),^43^ Genomics England,^44^ OpenSAFELY,^45^ UK Longitudinal Linkage Collaboration,^46^ UK Biobank,^47^ AllOfUS,^48^ Centers for Medicare & Medicaid Services (CMS),^49^ and Veterans Affairs Informatics and Computing Infrastructure (VA VINCI).^50^ The majority of these sources include electronic health records that contain sensitive patient information.

In a recently published research article, Deflaux et al.^51^ evaluated how different TREs influence cross-cohort analysis. They used two different well-known TREs, Research Workbench (AoU RW) from *All of Us* and Research Analysis Platform (UKB RAP) from UK Biobank and performed a genome-wide association study (GWAS) on circulating lipid levels, based on whole genome sequence data (from AoU RW) and whole exome sequence data (from UKB RAP). By performing GWAS twice—two meta-analyses from separate TREs—and performing pooled-analysis after combining the data, they reported that the two methods lead to similar but not identical results, especially for populations that are of non-European lineage. The key point of this study is to explore and compare different methodologies, namely meta-analysis and pooled data analysis, within the context of varying TREs.

There is a movement to create a larger, federated TRE designed to pool data from existing TREs. Torabi et al.^52^ introduced a six-tiered framework called Federated Health Data (FED-HD) governance, where each tier represents a level of data governance readiness for a centralized TRE to consolidate data from different repositories. For example, Tier 1 signifies separate TREs with no federation, Tier 3 allows sharing aggregated data and running federated queries, and Tier 6 represents the ultimate stage where data from diverse TREs are hosted in a global, federated network, enabling users worldwide to access and analyze the data.

Despite the many benefits of using TREs, because they require a DUA between institutions, there are administrative and legal burdens that can delay the data use process. Brophy et al.^53^ report that TREs rely on Data Access Agreements, which sometimes can be overly lengthy, complex, and outdated and suggests the need for a standardized cross-sectoral data access agreement template.

## Bringing Models to Data

### Model-to-Data Approach

The model-to-data approach, as its name suggests, brings the models to the location of the data and eliminates the need to transfer data. This approach puts an emphasis on the reversed flow of conventional information between data generators and data modelers, where data generators traditionally provide the data to the modelers.^54^ Two technologies, container software and cloud computing, have enabled the model-to-data to be used in real-world applications. A software container is a stand-alone unit of software that includes all the important elements, such as code and all dependencies and frameworks, to run in any environment. Tools like Docker allow users to create and manage software containers. Cloud computing allows users to use computing resources over the internet, which provides benefits for scalability and cost-efficiency. The model-to-data approach can use these technologies as models can be easily transported using container software and run in the cloud environment.^54^

By transferring models between data in different locations, the models can effectively learn from previously unseen, new data. This concept has been successfully implemented in crowdsource competitions^54^ like the Digital Mammography DREAM Challenge—where teams submitted contained programs to train models on unseen training data and was validated in new unseen data provided by Kaiser Permanente.^55^

Outside of the crowdsource settings, there are applications of this model in academic research settings. Mehta et al.^56^ performed a proof-of-concept study that showed that transferring models can effectively execute deep learning without any transfer of imaging data between two institutions. The model was trained to segment intraretinal fluid in structural OCT images of patients with diabetic macular edema, retinal vein occlusions, or macular degeneration at the University of Washington. The model was then transferred and used to segment intraretinal fluid in the OCT images of patients with exudative age-related macular degeneration from the New England Eye Center. The resulting model performance was comparable to that of human graders assessed with Dice coefficients or intersection over union scores.^56^^,^^57^

One significant advantage of using a model-to-data approach is the elimination of data transfer between data providers and modelers. This not only enhances patient information privacy but also reduces the legal complexities associated with data sharing agreements and dataset ownership. However, there are certain drawbacks to this method. During the initial stages of model development, it can be challenging to verify whether the model will operate with the format of the unseen data it will eventually be trained on. This includes considerations such as differing expected data types and variable names. To mitigate this challenge, it is essential to have access to examples that share the same format as the target dataset. An important step to make model-to-data feasible for collaborators or model developers would be having prior agreement on the data standards that model will be based upon. This could also be one of the reasons why the model-to-data format has had successful outcomes for data science challenges in the past as typical data science challenges have pre-agreed upon data formats and specific aims for the model to achieve. In practice, different institutions have different formats of data, and it is therefore necessary to harmonize inputs for collaborative learning ahead of time. It is crucial to note that this principle is not unique to the model-to-data approach but can generally be applied to all data sharing models. Harmonization of data standards by the contributing centers is essential in making data ready for collaborations. Additionally, for modelers, the lack of direct access to the datasets can limit the insights that can be gained through dataset exploration, which can be valuable for building an effective model. Another important consideration in the context of data provisioning is that the model developer may not have information about the computing resources that will be available to run the models.

### Federated Learning

**Figure 4.**
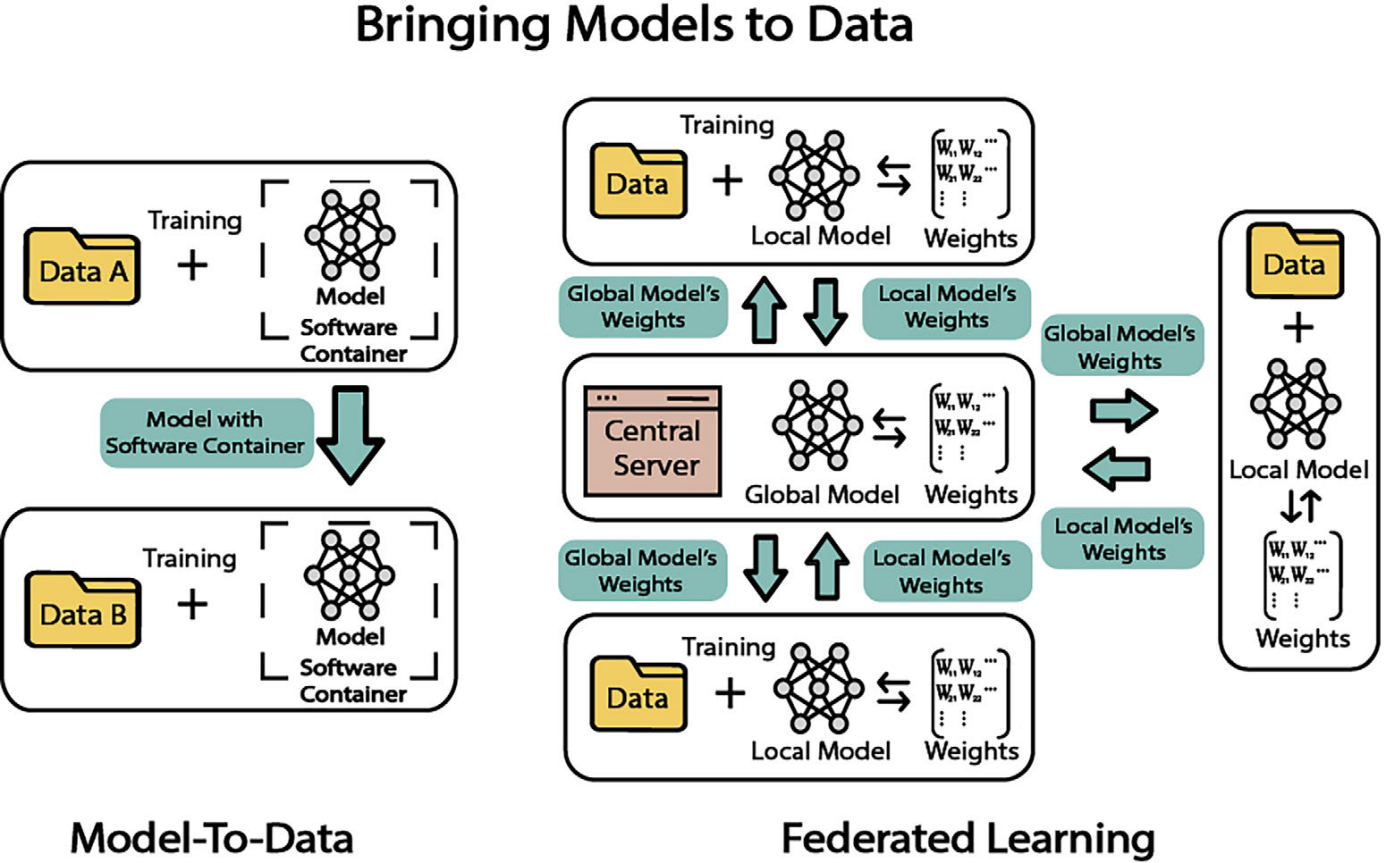
Model-to-data and federated learning.

In contrast to traditional models, where data from different sites is pooled together for centralized training, federated learning is a paradigm shift in machine learning that leverages distributed data sources, allowing for decentralized and privacy-protecting model development. Federated learning is a machine learning training setting where different participants update the algorithms collaboratively on a central server by sharing model weights instead of sharing each participant's data.^58^^,^^59^ It was initially introduced by Google in 2016.^60^ The workflow begins with the distribution of an initial model to each participant. These participants then independently train this model on their local data, a process that allows for diverse data inputs and personalized model adjustments. As shown in Figure 4, following local training, the updated model weights (not the data) are sent back to a central server for model aggregation. This stage involves combining the weights from each local model to form a global model. The updated global model is then redistributed to the participants for another round of local training. This process of model distribution, local training, and aggregation is iterative, ensuring that the global model gradually improves with each cycle. Throughout this iterative training, convergence needs to be evaluated to track the progress and performance of the model, ensuring that the learning is occurring. The process continues until a predetermined termination condition is met, such as a specific level of model accuracy. The traditional learning setting involves pooling all data from different sites together at a central location, where it is then trained collectively to obtain a model optimized for the aggregated data. In contrast, federated learning is a paradigm shift in machine learning that leverages distributed data sources, allowing for decentralized and privacy-protecting model development.

Federated learning reduces legal and administrative barriers to collaboration by eliminating the need for data use agreements, which are commonly required for healthcare data, because no data transfer occurs. However, it can require more computational resources, especially regarding communication.

Because local training needs to occur on multiple occasions, each participating site should have enough computing resources to process large data sets, and train a model on a local server iteratively. Additionally, model weights must be exchanged with the central server, which requires high bandwidth and high-speed networks to ensure timely updates. For cases where federated learning is done on mobile devices, which are not continuously connected to power, it is essential to have devices always charged and to ensure that user experience is not negatively affected by constant power usage for federated learning. Allowing asynchronous updates (when there is enough network bandwidth and power) and using smaller, simpler models can help alleviate these problems.

Blockchain and swarm learning are more modern technologies worth introducing for their new capabilities and its potential synergy with federated learning. Blockchain is a particular type of database consisting of a network of authenticated members or nodes which stores immutable information blocks that can be safely exchanged without interference by third parties, providing a tamper-proof record of transactions.^61^ A systematic review by Qammar et al.^62^ describes how blockchain could be used in federated learning applications. Adding block chain to federated learning enables traceability, by providing a record of the history of model updates, and decentralization, where multiple decentralized servers store model updates instead of centralized servers, which will be more resistant to a single point of failure attack. A newer technology that has some shared traits with federated learning but is distinct is swarm learning. Similar to federated learning, swarm learning enables models to be trained locally on different nodes, which can be servers or devices. However, unlike federated learning, these nodes function independently and do not send updates to the central server.^63^ Instead, the nodes share updates directly among themselves using the swarm network. Blockchain technology is utilized in swarm learning so that only authorized participants can perform and share updates. This can be helpful in the healthcare setting when collaborative learning is needed but confidentiality must be maintained. Warnat-Herresthal et al.^63^ demonstrated the feasibility of Swarm learning to develop disease classifiers, such as tuberculosis classification from blood transcriptomes.

Federated learning can be categorized into various types and variations based on certain criteria. First, based on the dataset characteristics, there are two main types: vertical and horizontal.^64^ Vertical federated learning is used when different participating groups have different features from the same data samples, making it ideal for collaborative learning across organizations that have overlapping samples but collect different types of information. In contrast, horizontal federated learning applies when different participating groups have similar features but from different samples, suitable for scenarios where data is fragmented across different regions or organizations. Furthermore, federated learning can be categorized based on the algorithm used for training, Some of the popular methods include federated averaging (FedAvg),^60^ which involves averaging local updates to improve the global model, and federated proximal gradient descent (FedProx), which introduces a regularization mechanism and is designed to handle non-identical distributions (statistical heterogeneity).^65^ Other variations include FedMRL,^66^ FedSR,^67^ FedSAM,^68^ which mainly focused on enhancing model generalization.

In the field of ophthalmology, federated learning has been applied to different tasks, mainly imaging related. Lo et al.^69^ and Yu et al.^70^ applied this approach to diabetic retinopathy classification and microvasculature segmentation of OCTA/OCT en face images, utilizing residual VGG and U-Net architectures, respectively. Similarly, Lu et al.^71^ classified retinopathy of prematurity in federated learning fashion on fundus images collected from seven institutions using a ResNet-18 model. These studies used the method of averaging the model weights to perform the global model updates and showed that federated learning method was non-inferior to using aggregated data. More recent studies investigating glaucoma related disease^72^^,^^73^ have used FedProx^65^ to make each client's model update close to the initial global model. A recent federated learning study used both CNN architecture, ResNet18, and a vision transformer (ViT) to diagnose age-related macular degeneration from OCT scans using three separate datasets (simulating three institution settings).^74^ The authors investigated different federating learning methods for different purposes, including FedProx to address data heterogeneity, FedSR to simplify model's representation, and FedMRI to address domain shift.

Federated learning offers several advantages over traditional centralized learning approaches where data from different sites is pooled together, in terms of privacy and efficiency. Additionally, data ownership is preserved; each participant retains control over their own data, which is not shared or transferred. This aspect is crucial in scenarios where data privacy is paramount. Another significant advantage is the reduced need for data transfer. In federated learning, massive datasets do not need to be centrally stored or processed, which not only saves bandwidth but also reduces the risks associated with data transmission.

However, federated learning also comes with its set of challenges. One such challenge is the presence of stragglers, or participants that take longer to complete their updates due to lack of computing resources or poor connectivity, potentially delaying the overall training process.^75^ Another significant challenge is model combining. Creating a cohesive and effective global model can be difficult when dealing with heterogeneous data from diverse sources, each with potentially different distributions and characteristics. This heterogeneity often requires more sophisticated approaches to model aggregation.^65^ Furthermore, federated learning typically involves more complex computation and communication compared to centralized learning. Managing and synchronizing updates across numerous devices, each with potentially different data structures and computing capabilities, adds more complexity to the learning process, both in terms of computation and communication process. One challenge for federated learning is its vulnerability to white box attacks, which is a scenario in which a cyber-attacker has knowledge of the system, such as model parameters and architecture.^76^ Nasr et al.^77^ reported that neural networks are also susceptible to different inference attacks in a federated learning setting from either the central server or one of the participants. A central server can collect updates from each participant and acquire information about their data details. On the other hand, a participant can observe how the global parameters change over time and send crafted updates to the central server in order to learn more about other participants’ data. In both cases, there is a risk that an entity with malicious intent can use the system to their advantage, highlighting the need to recognize that federated learning platforms can be vulnerable to white box attacks.^77^ Last, because of the collaborative nature of federated learning, it can be subject to local, client-side model theft or model data leakage. Intellectual property schemes such as watermark-based methods have been proposed to protect data ownership.^78^^,^^79^

## Discussion

It is important to note that none of the data sharing methods discussed here are mutually exclusive and can be combined to create a mixed-method approach. For instance, federated learning can be used using synthetic data.^80^ Similarly, differential privacy techniques can be applied within a federated learning setting.^81^ Data collected for open-source datasets, although ultimately intended for public availability, must first be securely stored in a platform. This step is essential until the data are fully anonymized, processed, and standardized.

Data sharing methods may influence machine learning model performance, with variability depending on factors such as model architecture, data types, data quality, and training setup. For instance, studies in federated learning often strive to demonstrate comparable performance to centralized training, showing that accuracy need not be sacrificed in the federate learning setting.^69^^,^^71^ The use of synthetic data, often combined with original datasets, has been found to enhance model performance in various studies. This better performance could be due to the increased diversity from synthetic datasets, which can help generalize the model. However, the incorporation of differential privacy measures, such as noise that aims to protect individual data points may introduce variability in model performance. While some studies have demonstrated maintained or even non-inferior performance levels for specific tasks, others have observed performance declines, particularly affecting underrepresented groups within the datasets.^27^

Trusted research environments and model-to-data strategies put emphasis on securing the location of the data, focusing less on the direct implications of privacy measures on the training process. The effect of each data sharing method on model accuracy is highly dependent on the specific needs and design of the project. Researchers are therefore required to select the suitable data sharing methods that align with both the intended application of the model and the privacy requirements. This selection process is important in ensuring both the performance and ethical considerations of research projects.

In Table 2, we present a comparison of newer approaches to data sharing. This analysis evaluates methods for sharing data discussed in this review, highlighting their differences in terms of mechanism, data access control, data modification, real data, and adjustment of privacy parameters. Each approach serves different purposes and comes with its own set of advantages and limitations. Understanding these differences is crucial for researchers to choose the suitable method based on their specific requirements for data sharing, privacy, and analysis.

**Table 2.** Comparison of Newer Approaches to Data Sharing

	Differential Privacy	Synthetic Datasets	Trusted Research Environment	Model-to-Data Approach	Federated Learning
Security Mechanism	Noise is added to data	Synthetic data is not real data	Authorized users can access data and do analysis in a secure location	No data transfer occurs but model is transferred	No data transfer occurs but model weights are transferred
Data Access Control	No	No	Yes	No	No
Data Modification	Yes	Yes	No	No	No
Real Data	Yes	No	Yes	Yes	Yes
Adjustment of Privacy Parameters	Yes	Depends	No	No	Depends

In this review, we have explored the spectrum of data sharing methods applicable to the field of AI in ophthalmology, with a special focus on aiding clinicians in understanding and utilizing these methods effectively. AI is becoming increasingly integrated into a wide range of medical research and practice, including diagnosis assistance, treatment recommendation, and patient monitoring. While the mainstream research focus has been on developing high performing AI models, the significance of data sharing methods will only grow over time, as the quality and performance of all models inherently rely heavily on their training data. Associated challenges in ethics, security, and policies will inevitably arise and need to be addressed. As AI continues to influence healthcare, choosing safe and effective data sharing methods will be of importance to protect sensitive information and advance medical research.
